# Expert Evaluation of ChatGPT-4 Responses to Upper Tract Urothelial Carcinoma Questions: A Prospective Comparative Study with Guideline-Based and Patient-Focused Queries

**DOI:** 10.3390/jcm14186353

**Published:** 2025-09-09

**Authors:** Murat Beyatlı, Hasan Samet Güngör, Abdurrahman İnkaya, Resul Sobay, Ahmet Tahra, Eyüp Veli Küçük

**Affiliations:** Department of Urology, Umraniye Training and Research Hospital, 34764 Istanbul, Turkey; drsametgngr@gmail.com (H.S.G.); ainkaya@hotmail.com (A.İ.); drresulsobay@gmail.com (R.S.); ahmettahra@gmail.com (A.T.); eyupveli@gmail.com (E.V.K.)

**Keywords:** artificial intelligence, ChatGPT, clinical guidelines, upper urinary tract, urothelial carcinoma

## Abstract

**Background/Objectives:** This study aimed to systematically evaluate the accuracy and clinical relevance of ChatGPT-4’s answers to a set of questions on upper tract urothelial carcinoma (UTUC) as adjudged by expert urologists. To juxtapose performance, one question set consisted of queries derived from the 2025 EAU (European Association of Urology) guidelines, and the other was a miscellaneous set of frequently asked patient-oriented questions. **Methods**: Seventy-seven questions were posed to ChatGPT-4 in English: 60 were systematically selected from the 2025 EAU UTUC guidelines, and 17 were compiled as frequently asked questions (FAQs) from reputable urology sources. Two board-certified urologists independently scored the given responses, employing (a) binary scoring (correct = 1, incorrect = 0) and (b) detailed accuracy scoring (1 = completely accurate, 2 = accurate but inadequate, 3 = mixed accurate–misleading, 4 = completely inaccurate). Comparative analyses used the Mann–Whitney U test with effect size estimation. **Results**: Overall, 71 of 77 responses (92.2%) were correct. Accuracy rates were 90.0% (54/60) for EAU guideline questions and 100.0% (17/17) for the FAQs. The mean accuracy score for the guideline-based questions was 1.28 ± 0.74, compared with 1.00 ± 0.00 for the FAQs. Differences between the groups were not statistically significant (*p* = 0.094, r = 0.191). A subgroup analysis showed perfect accuracy (100%) in four EAU categories—Classification and Staging Systems, Diagnosis, Disease Management, and Metastatic Disease Management—while the Follow-up category had the lowest accuracy (25% correct, mean score = 2.75), indicating domain-specific limitations. **Conclusions**: ChatGPT-4 demonstrated high overall accuracy, particularly for patient-oriented UTUC questions, but showed reduced reliability in complex, guideline-specific areas, especially follow-up protocols. The model shows promise as an educational tool for patients but cannot replace expert clinical judgment for decision-making. These findings have important implications for the integration of AI tools in urological practice and highlight the need for domain-specific optimization.

## 1. Introduction

Artificial intelligence (AI) has made great progress in the medical field within the last few years, bringing in an entirely new array of possibilities with respect to the presentation of medical information, clinical decision support, and patient education [1]. Large language models (LLMs), such as ChatGPT developed by OpenAI, are designed specially to produce fluent and contextually relevant human-quality responses to an extremely wide range of clinical questions through the use of advanced natural language processing techniques [2]. While technically these features have the power to increase access to quality health information, concerns exist about the validity, credibility, and integrity of AI-generated content in specialist medical subjects [3].

The integration of AI into health care represents a paradigm shift that extends beyond simple information retrieval to encompass complex clinical reasoning tasks. Modern LLMs demonstrate remarkable capabilities in understanding medical terminology, interpreting clinical scenarios, and generating coherent explanations that mirror human expertise. However, the transition from general medical knowledge to subspecialty-specific applications requires careful evaluation to ensure patient safety and clinical utility.

Upper urinary tract urothelial carcinoma (UTUC) is a rare but aggressive tumor, accounting for about 5–10% of all urothelial carcinomas [4]. Diagnosing and managing UTUC is complicated, requiring multilevel imaging, histopathological assessment, and options for both surgical and systemic treatment [4,5]. The evolution of diagnostic modalities, including high-resolution computed tomography urography, ureteroscopy with biopsy, and workup involving molecular profiling, has improved capabilities for early detection; yet the last frontier of large randomized controlled trials being sparse means that most clinical decision-making is grounded predominantly in expert consensus. Clinicians as well as patients require prompt access to accurate evidence-based data. The European Association of Urology (EAU) guidelines form the gold standard for the treatment of UTUC, providing organized recommendations in the areas of epidemiology, diagnosis, risk stratification, treatment, and follow-up [4].

Several online medical databases have been growing in popularity over the years, with the onset of COVID19 making this spike even more noticeable [6,7,8]. Hence, AI-based platforms are gaining prominence for patient engagement and professional education alike. The newest generation of OpenAI’s LLMs, ChatGPT-4, has an improved reasoning and contextual understanding ability over its predecessors [9]; however, it has to be seen about its application in specialized domains, such as UTUC. Previous studies have assessed LLMs in general medical examinations and in certain urological contexts, but comprehensive evaluations in UTUC, particularly direct comparisons between guideline-based and patient-focused questions, are lacking [10,11,12]. Understanding performance differences across question types and clinical domains is important for defining the potential for and limitations of AI in clinical support.

This prospective comparative study evaluated ChatGPT-4’s performance on two distinct question sets: one systematically derived from the 2025 EAU UTUC guidelines and another composed of frequently asked patient-oriented questions from reputable urology sources. We hypothesized that ChatGPT-4 would achieve higher accuracy on general patient-focused questions than on highly specific, guideline-based clinical queries. By simultaneously evaluating ChatGPT-4 on EAU guideline-based and patient-focused question sets, our study addresses this knowledge gap and provides a dual-perspective analysis that is relevant to both clinicians and patients seeking reliable medical information.

## 2. Materials and Methods

This prospective comparative study was conducted in May–July 2025. Since this study did not include human participants, patient records, or experimental manipulation, and used exclusively publicly available data, formal ethics committee approval was not sought. The research conformed to the reporting guidelines of Strengthening the Reporting of Observational Studies in Epidemiology (STROBE) [13].

Two distinct categories of questions were used to assess ChatGPT-4’s performance:

- EAU Guideline-Based Questions (*n* = 60): Specific clinical questions were systematically extracted from the 2025 EAU Upper Tract Urothelial Carcinoma section. These questions covered explicit recommendations and in-text evidence statements on epidemiology, etiology, pathology, diagnosis, staging, risk stratification, treatment, metastatic disease management, and follow-up.
- Frequently Asked Questions (FAQs) (*n* = 17): General patient-oriented questions were compiled from major international urology association websites and reputable medical information portals. These questions reflected common, non-specialist inquiries frequently encountered in clinical consultations or online patient forums (Appendix A, Table A1).

Guideline-based questions were identified through a structured review of the full 2025 EAU UTUC guideline document by two independent reviewers. Each of the reviewers in turn culled possible items from sections on epidemiology, etiology, diagnosis, staging, risk stratification, treatment, management of metastatic disease, and follow-up, so that every question aligned to a clearly defined recommendation or evidence statement.

For patient-oriented FAQs, questions were systematically identified from multiple high-quality sources including the American Urological Association (AUA) patient education materials, the British Association of Urological Surgeons (BAUS) patient information resources, the Urology Care Foundation educational content, and peer-reviewed patient education publications. A standardized selection process was implemented using the following inclusion criteria: (1) questions commonly encountered in clinical practice based on expert consensus, (2) clearly phrased for lay audiences without excessive medical terminology, (3) directly relevant to UTUC diagnosis, treatment, or prognosis, and (4) representative of typical patient concerns and information-seeking behaviors.

All questions were posed to ChatGPT-4 (OpenAI, San Francisco, CA, USA) in English on 31 May 2025. Each question was asked individually in a fresh chat session to avoid contextual carry-over. Original responses were recorded without alteration. The model version from 31 May 2025, was used for evaluation.

Two board-certified urologists, each with over a decade of clinical experience, independently reviewed the AI-generated responses. Both urologist reviewers were blinded to the category of each question during evaluation to minimize bias. Disagreements in scoring were resolved through direct discussion, achieving full consensus without the need for a third adjudicator. The evaluation utilized two scoring systems as follows:

- Binary Answer Scoring:

1 = Correct

0 = Incorrect

- Detailed Accuracy Scoring:

1 = Completely correct

2 = Correct but inadequate

3 = Mixed correct and misleading information

4 = Completely incorrect

Data analysis was performed using Python 3.9 (Python Software Foundation, Wilmington, DE, USA) and SPSS 28.0 (IBM Corp., Armonk, NY, USA). Categorical variables were expressed as frequencies and percentages, while continuous variables were presented as mean ± standard deviation (SD). Group comparisons employed the Mann–Whitney U test. The effect size was calculated as Cohen’s r. Statistical significance was set at *p* < 0.05.

For enhanced interpretability, performance was further stratified by EAU guideline category, and error type distributions were analyzed to identify patterns of AI performance variability.

## 3. Results

Out of 77 questions, ChatGPT-4 provided correct responses to 71, yielding an overall accuracy rate of 92.2% (95% CI: 84.60–96.4%). The accuracy rate for EAU guideline-based questions was 90.0% (54/60) (95% CI: 79.9–95.3%), compared with 100.0% (17/17) (95% CI: 81.6–100%) for the FAQs (Table 1). All six incorrect responses were from the EAU guideline-based set.

**Figure 1:**
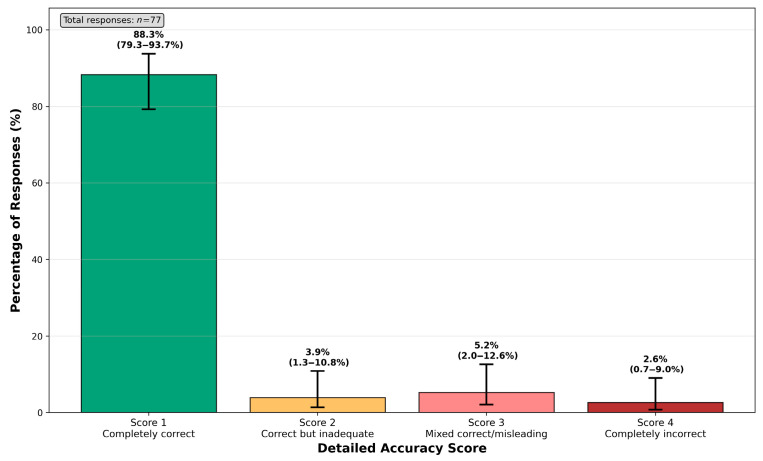
Detailed accuracy scoring distribution.

In the detailed accuracy scoring, 88.3% (68/77) of responses were rated as completely correct (score = 1), 3.9% (3/77) as correct but inadequate (score = 2), 5.2% (4/77) as mixed correct/misleading (score = 3), and 2.6% (2/77) as completely incorrect (score = 4) (Figure 1).

The mean accuracy score for EAU guideline-based questions was 1.28 ± 0.74, whereas all FAQ responses achieved a perfect mean score of 1.00 ± 0.00. A statistical comparison using the Mann–Whitney U test showed no significant difference between groups (*p* = 0.094), with a small effect size (Cohen’s r = 0.191).

**Figure 2:**
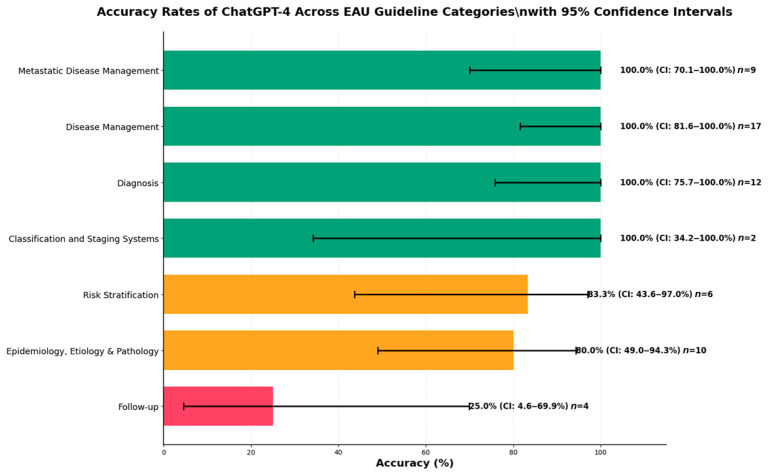
Accuracy rates of ChatGPT-4 across EAU guideline categories. Green bars indicate categories with ≥90% accuracy, orange bars indicate moderate accuracy (50–89%), and red bars indicate low accuracy (<50%).

A subgroup analysis by EAU guideline category demonstrated substantial variation in performance (Figure 2). Four categories—Classification and Staging Systems, Diagnosis, Disease Management, and Metastatic Disease Management—achieved perfect accuracy (100%). Moderate performance was noted for the categories Epidemiology, Etiology & Pathology (80.0%), and Risk Stratification (83.3%). The lowest accuracy was seen in the Follow-up category, with only 25.0% correct responses and a mean accuracy score of 2.75.

The figure highlights strong performance in diagnostic and management domains and a pronounced weakness in follow-up-related questions.

## 4. Discussion

This study represents the first comprehensive evaluation of ChatGPT-4’s performance in UTUC using a structured comparison between EAU guideline-based clinical questions and frequently asked patient-oriented queries. The findings show that, while the model performs exceptionally well in general and commonly discussed areas, its accuracy declines notably in specialized categories, most prominently in follow-up-related questions. These results have important implications for the clinical integration of AI tools and highlight both the promise and limitations of current language models in subspecialty medical domains.

The observed performance pattern reflects the fundamental architecture of large language models, which excel at retrieving and synthesizing information that is well-represented in their training data but struggle with highly specialized, protocol-driven, or recently updated clinical knowledge [9]. This finding is particularly relevant for UTUC, where management decisions often rely on complex risk stratification algorithms and institution-specific protocols that may not be widely disseminated in the medical literature.

The overall accuracy rate of 92.2% aligns with previous studies evaluating ChatGPT in other medical fields. Recent studies have shown varying performance rates for different ChatGPT versions on medical examinations. For instance, earlier evaluations of ChatGPT-3 on USMLE questions demonstrated accuracy rates ranging from 44% to 64% depending on the specific examination component [14], while more recent assessments using ChatGPT-4 have shown improved performance, and Garabet et al. found an 86% success rate in a similar evaluation [15]. The higher accuracy in our study, especially the 100% correct rate for the patient-oriented FAQs, suggests ChatGPT-4 is well-tuned for the general medical information commonly represented in its training data.

The non-significant *p*-value (*p* = 0.094) with a small effect size (r = 0.191) suggests our study may be underpowered for detecting meaningful differences between guideline-based and FAQ performance. However, the clinical relevance of this difference is minimal, as both groups demonstrated high accuracy rates (90.0% vs. 100.0%). The small effect size indicates that the practical difference between these groups is negligible for clinical decision-making purposes.

Studies assessing ChatGPT’s performance in urology use varied methods. A recent study evaluated ChatGPT-3.5 on UTUC using 15 patient-derived questions, scoring an average of 3.93 out of 5 from 16 experts [12]. By contrast, our study used 60 EAU guideline-based questions and 17 FAQs (77 questions in total), evaluated by two experts using binary scoring. The methodological differences between the studies underscore the need for standardized evaluation frameworks for AI performance in medical domains. Our binary scoring approach, while simpler, provides clear clinical decision points that may be more relevant for practical implementation.

Comparative studies have noted a performance gap between ChatGPT-3.5 and ChatGPT-4 in urological question answering. For instance, ChatGPT-3.5 scored lowest in the treatment category (3.68/5.0), whereas ChatGPT-4 demonstrated substantially higher performance here, achieving perfect accuracy (100%) in patient-focused questions and excelling across several guideline-based domains [12]. These results indicate that newer model versions improve medical accuracy, especially in specialized areas like uro-oncology, highlighting the benefit of leveraging updated AI models while maintaining expert oversight.

Despite high overall accuracy, the 10% error rate in guideline-based questions and the presence of partially correct or misleading information reveal an important limitation: the model’s vulnerability in specialized, less common clinical scenarios. This aligns with evidence that LLMs perform best on frequent, generalizable content and less reliably on rare, nuanced, or recently updated topics [16].

A category-level analysis offers insight into ChatGPT-4’s strengths and weaknesses. Perfect accuracy was reached for the categories Classification and Staging Systems, Diagnosis, Disease Management, and Metastatic Disease Management, domains that are conceptually established and well represented in the medical literature. These categories typically involve factual medical knowledge with clear diagnostic criteria and established treatment algorithms that have remained relatively stable over time, and are extensively covered in medical education materials.

Moderate accuracy in the categories Epidemiology, Etiology & Pathology (80.0%), and Risk Stratification (83.3%) suggests that ChatGPT-4 can retrieve foundational knowledge but struggles to integrate recent epidemiologic data or risk-based decision algorithms. The epidemiological errors were particularly concerning as they involved citing outdated incidence rates and survival statistics that could mislead patients about their prognosis. This highlights a fundamental limitation of static training datasets that cannot incorporate the most recent clinical research and population-based studies.

The most notable finding is the markedly low accuracy in the Follow-up category (25.0%), accompanied by the highest proportion of completely incorrect responses. This likely reflects the complexity and variability of follow-up protocols, which often require adjustments tailored to patient characteristics, comorbidities, and institutional practices. These protocols are also frequently updated in guidelines, factors possibly underrepresented in the model’s static training data. The clinical implications of these follow-up errors are particularly serious, as inadequate surveillance could lead to delayed detection of recurrence or progression, while excessive surveillance could result in unnecessary radiation exposure and healthcare costs.

The clinical implications of errors in follow-up protocols are particularly concerning. Incorrect surveillance schedules could lead to delayed recurrence detection, potentially missing the optimal window for salvage therapy. For example, if ChatGPT-4 recommends an inadequate imaging frequency for high-risk patients, recurrent disease might progress to an unresectable stage before detection. Conversely, overly aggressive surveillance recommendations could expose patients to unnecessary radiation, increase healthcare costs, and cause psychological distress. These risks underscore why AI-generated follow-up recommendations must always be verified by specialists familiar with individual patient risk factors, comorbidities, and institutional protocols.

An error type analysis showed that ChatGPT-4’s errors vary. Most errors in higher-performing categories were “correct but inadequate” or “mixed correct/misleading”, which may retain some clinical value if reviewed by an expert. However, in the Follow-up category, the significant number of completely incorrect answers raises patient safety concerns if used without expert review. These findings underscore the critical importance of implementing appropriate safeguards and expert oversight when integrating AI tools into clinical workflows.

The integration of AI tools like ChatGPT-4 into clinical practice raises complex medico-legal questions about accountability and responsibility. Currently, healthcare providers remain legally responsible for all clinical decisions, regardless of AI input. If AI-provided information contributes to adverse outcomes, the attending physician bears primary liability, highlighting the need for thorough verification of AI-generated content. Healthcare institutions must establish clear policies regarding AI tool usage, including mandatory expert review protocols and documentation requirements. Furthermore, patients should be informed when AI tools contribute to their care and education, ensuring transparency in the clinical decision-making process. As AI becomes more prevalent, regulatory frameworks must evolve to address these accountability challenges while preserving the benefits of AI-assisted care.

These underline the necessity of human verification in the use of AI instruments in clinical practice, especially in cases of complex and individualized management of the patient.

From a technical standpoint, LLMs like ChatGPT-4 are trained on broad datasets with limited direct exposure to specialty-specific guidelines such as the EAU UTUC recommendations [17,18]. While the model’s reasoning enables approximating guideline-consistent answers, its lower accuracy in follow-up and risk-based questions suggests that domain-specific fine-tuning or real-time guideline integration is necessary for optimal clinical use [18].

Based on our results, we propose the following recommendations for ChatGPT-4 integration in urology:

- Safe Use for Patient Education: The model reliably provides general disease information to patients, especially for FAQs, but should always include disclaimers about the need for professional medical consultation.
- Caution in Clinical Decision Support: Guideline-based and complex management questions should always be verified by experts, particularly in areas involving follow-up protocols and risk stratification.
- Targeted Model Optimization: Incorporating updated guideline datasets and supervised fine-tuning may improve weaker areas, with particular focus on dynamic clinical protocols and recent research findings.
- Ongoing Monitoring: Regular reassessment is essential as AI models and clinical guidelines evolve, with systematic evaluation protocols to track performance over time.

Healthcare institutions considering AI integration should develop comprehensive governance frameworks addressing training requirements, oversight protocols, liability considerations, and quality assurance measures. These frameworks should include regular performance audits, user feedback mechanisms, and clear escalation pathways for complex cases.

The integration of AI tools into clinical practice necessitates corresponding changes in medical education and training. Healthcare professionals must develop competencies in AI literacy, including an understanding of model limitations, appropriate use cases, and quality assessment techniques. Medical curricula should incorporate training on AI tools while emphasizing the irreplaceable value of clinical judgment and expert knowledge.

Artificial intelligence methods have become more and more incorporated in medicine. Such models as Bidirectional Encoder Representations from Transformers (BERT), trained first on general collections, show high performance after tuning on domains of specialization [19]. In urology, targeted fine-tuning may create models better suited for complex, domain-specific questions. Recent work shows that LLMs can encode substantial clinical knowledge; when combined with real-time guideline updates and expert feedback, accuracy and relevance improve significantly [20].

Patient education programs should also evolve to help patients understand both the benefits and limitations of AI-generated health information. Patients should be empowered to critically evaluate AI-generated content and to understand when professional medical consultation is essential.

Finally, the larger ethical concerns of applying AI to patient and professional settings must be reckoned with. Though our results affirm the utility of ChatGPT-4 as a complementary information resource, there exists the danger of overdependence should users mix up fluency and factual correctness. Ongoing medical education of professionals, focusing on AI tool strength and weakness, will be necessary to avert misuse. Beyond that, AI builders, clinical specialists, and regulatory authorities working together could engender specialized, periodically updated models to handle the distinctive informational requirements of high-risk, low-incidence diseases like UTUC.

The economic implications of AI integration in health care are substantial, with potential benefits including improved efficiency, reduced healthcare costs, and enhanced access to medical information. However, these benefits must be balanced against implementation costs, training requirements, and the need for ongoing quality assurance. Healthcare systems should conduct comprehensive cost-benefit analyses before implementing AI tools in clinical workflows.

It is also worth noting that our evaluation, while prospective and structured, did not attempt to measure the interpretability or transparency of the model’s reasoning process. Future research could include qualitative examination of the rationales produced by the model, not simply checking if a solution is right, but if the justification follows logically, has evidence to back it, and makes sense to the end user. This dimension of evaluation could be critical in determining clinician adoption and patient trust.

From a healthcare system perspective, these findings raise questions about the optimal integration of AI tools into multidisciplinary workflows. For example, a urologist could use ChatGPT-4 to generate a preliminary patient education summary, which would then be reviewed and edited for clinical accuracy. Such a model could streamline communications while safeguarding against the propagation of errors. In parallel, policy-level interventions—such as mandating provenance tracking for AI-generated clinical content—could enhance trust and accountability.

Several important limitations must be acknowledged when interpreting these results. First, our evaluation was limited to two expert reviewers, which, while achieving excellent inter-rater agreement, may not capture the full spectrum of clinical opinion that would be obtained from a larger, more diverse panel of experts. The relatively small sample size of questions, particularly in individual EAU categories, limited our ability to detect subtle performance differences and may have reduced the statistical power of the subgroup analyses.

Our single-turn evaluation design, while ensuring standardization, does not reflect real-world clinical interactions where follow-up questions and iterative clarifications are common. In clinical practice, AI tools are more likely to be used in conversational contexts where additional patient details, imaging findings, or clarifying questions might improve response accuracy. Future research should evaluate ChatGPT-4’s performance in multi-turn, context-rich dialogues to better assess its practical clinical utility.

The linguistic and cultural limitations of our study also deserve consideration. All questions were posed in English, and the evaluation was conducted by experts trained in Western medical traditions. Performance may differ significantly in other languages or when addressing region-specific clinical practices and patient populations.

The performance gap within the Follow-up category deserves specific attention when framed in terms of clinical risk. Follow-up schedules in UTUC are not only heterogeneous across institutions but subject to rapid evolution, such as imaging modalities, risk stratification tools, and cost-effectiveness data evolution. A language model that does not have access to current advances in the literature is consequently lost in developing modern and correct advice. This limitation could be mitigated through hybrid systems in which AI-generated content is automatically cross-referenced with the latest guideline repository prior to delivery to the end user.

Another relevant factor relates to the wording and construction of the questions themselves. Guideline-based questions in our dataset were often concise and directly aligned with EAU recommendations, but they still required the model to recall or infer precise details. By contrast, the patient-oriented FAQs typically framed concepts in broader, more conversational language. Such differences in linguistic framing may inherently favor the model, given that large language models are predominantly trained on conversational and general-domain text. This would indicate that future tests should try out questions of differing formats, including intentionally unclear or multi-component questions, to better evaluate the depth of reasoning capabilities.

In interpreting these results, it is also important to consider that the high performance observed in several categories may be partially driven by the overlap between widely disseminated clinical knowledge and the model’s training corpus. Queries based on universally accepted treatment or diagnostic paradigms, such as CT urography for diagnosis or radical nephroureterectomy for high-risk disease, would more likely align with probabilistic predictions of the model. This phenomenon may inflate apparent accuracy in these domains while obscuring potential weaknesses in areas requiring dynamic, case-specific reasoning.

Our study had several limitations, as acknowledged above. First, all questions were in English, leaving performance in other languages untested. Second, this study focused on a single disease, UTUC, so findings may not generalize to other urological cancers. Third, the evaluation involved only two experts, though consensus scoring helped to reduce bias. Fourth, the single-turn evaluation design may not reflect real-world clinical interactions where clarification and follow-up questions are common. Fifth, our study was potentially underpowered for detecting smaller effect sizes in subgroup comparisons. Sixth, the model version reflects its state as of 31 May 2025; future updates may affect results. Finally, real-world clinical practice often involves multi-turn dialogues and contextual factors that are not captured in this single-question evaluation design [21].

Future research priorities should include the following: (1) Multi-turn conversational evaluation to better reflect real-world usage patterns; (2) Comparative studies benchmarking ChatGPT-4 against medical trainees and specialists; (3) Multilingual evaluation to assess global applicability; (4) Domain-specific fine-tuning using current clinical guidelines; (5) Real-time integration with updated medical literature; (6) Evaluation across multiple cancer types and medical specialties; (7) Assessment of AI performance in complex clinical scenarios requiring multi-factorial decision-making; and (8) Long-term studies examining how AI integration affects clinical outcomes and physician decision-making patterns.

## 5. Conclusions

ChatGPT-4 showed high overall accuracy answering questions on UTUC, with especially strong performance on the patient-oriented FAQs. While accuracy remained high on guideline-based clinical questions, it was notably lower in the Follow-up domain, where a greater share of completely incorrect answers occurred. These findings provide important insights into the current capabilities and limitations of large language models in specialized medical domains and offer guidance for their safe and effective clinical implementation.

These findings indicate that ChatGPT-4 can be an adjunct to routine medical questions and patient education, but should not be used alone to guide complex clinical decisions. Expert supervision should be in place in situations concerning patient safety. The observed performance patterns reflect fundamental characteristics of current AI technology, with excellent performance on well-established medical knowledge but significant limitations in protocol-driven, dynamic, or recently updated clinical areas. Future improvements, such as domain-specific fine-tuning and real-time guideline updates, may close the current gaps and enhance the model’s role in specialized clinical settings.

Our findings are specific to UTUC and may not extrapolate to other urological cancers or broader oncological contexts. The unique characteristics of UTUC management may not represent the full spectrum of challenges in AI-assisted oncology care.

Healthcare institutions and individual practitioners considering AI integration should proceed with careful attention to governance, training, and quality assurance. The promise of AI in enhancing health care delivery is substantial, but realizing this potential requires thoughtful implementation that prioritizes patient safety and maintains the central role of clinical expertise.

## Figures and Tables

**Table 1:** Comparative analysis of ChatGPT-4’s performance.

Category	Correct Answers *n* (%)	Incorrect Answers *n* (%)	Accuracy Score Mean ± SD	Accuracy Score Median (IQR)	95% CI
EAU Guidelines (*n* = 60)	54 (90.0)	6 (10.0)	1.28 ± 0.74 *	1.0 (1.0–1.0)	79.9–95.3%
Frequently Asked Questions (*n* = 17)	17 (100.0)	0 (0.0)	1.00 ± 0.00 *	1.0 (1.0–1.0)	81.6–100%
Total (*n* = 77)	71 (92.2)	6 (7.8)	1.22 ± 0.66	1.0 (1.0–1.0)	84.0–96.4%

EAU: European Association of Urology. * *p* value (Mann–Whitney U Test): 0.094; Effect size (Cohen’s r): 0.191.

## Data Availability

The data presented in this study are available on request from the corresponding author.

## Abbreviations

The following abbreviations are used in this manuscript:

EAU	European Association of Urology
UTUC	Upper Tract Urothelial Carcinoma
FAQs	Frequently Asked Questions
LLM	Large Language Models
STROBE	Strengthening the Reporting of Observational Studies in Epidemiology
USMLE	United States Medical Licensing Examination
BERT	Bidirectional Encoder Representations from Transformers

## Appendix A

**Table A1:** Detailed Clinical Questions Analyzed in this Study.

Issue	Question to ChatGPT
Epidemiology Etiology and Patology	Is there a relationship between aristolochic acid and upper urinary tract carcinoma?
	Is upper urinary tract carcinoma associated with any syndrome?
	What criteria are used to evaluate Lynch syndrome in patients with upper urinary tract carcinoma?
	What are the Amsterdam 2 criteria?
	What tests should be performed in patients with suspected hereditary upper urinary tract urothelial carcinoma?
	What is the prevalence of UTUC in high-risk NMIBC patients treated with intravesical bacillus Calmette–Guérin (BCG)?
	What is the prevalence of UTUC in patients with MIBC treated with radical cystectomy?
	What is the risk of bladder recurrence in UTUC patients after UTUC treatment?
	What percentage of patients have muscle invasion at the time of diagnosis?
Classification And Staging Systems	Which patients with upper urinary tract urothelial carcinoma should be offered mismatch repair (MMR) proteins or microsatellite instability testing? Which staging system is used for upper urinary tract urothelial cell carcinoma? Which grading system is used for upper urinary tract urothelial cell carcinoma?
Diagnosis	Which surgical or imaging method should be used in the diagnosis and staging of upper urinary tract carcinoma?
	What can voided cytology indicate when cystoscopy is normal and there is no CIS in the bladder and prostate urethra?
	What is the sensitivity of selective urine cytology in urothelial high-grade tumors, including carcinoma in situ? Should cystoscopy be performed in upper urinary tract tumors?
	Why should cystoscopy be performed in upper urinary tract tumors? Which patients with suspected upper urinary tract tumors should we perform cytology? Which imaging technique should be used for the diagnosis and staging of upper urinary tract tumors?
	Which imaging technique should be used for chest imaging in high-risk upper urinary tract tumors?
	Which imaging modality should be used to exclude metastases in patients with high-risk upper urinary tract tumors? Should urethral ureteroscopy be performed for diagnosis and/or risk stratification in every patient with suspected upper urinary tract urothelial carcinoma? In which patients with upper urinary tract tumors should we perform FGFR 2/3 testing? Is the risk of thoracic metastasis low or high in low-risk UTUC?
Risk Stratification	For nonmetastatic UTI, what factors are included in the risk stratification based on risk of progression to >pT2/non-organ-confined disease? Which factors are considered high risk?
	What are the factors affecting the risk of bladder recurrence after surgery for Upper Urinary Tract Urothelial Cell Carcinoma?
	Are there any validated molecular biomarkers for clinical use in Upper Urinary Tract Urothelial Cell Carcinoma? Approximately how many UTUC patients have multifocal tumors?
	Does hydroureteronephrosis affect the prognosis in patients treated with RNU? What are the strong and weak criteria when we look at the high-risk group in UTUC?
Disease Management	What is the primary treatment option for low-risk upper urinary tract tumors?
	What are the treatment options for low-risk tumors of the distal ureter?
	When should a second look ureteroscopy be performed after the first endoscopic treatment in upper urinary tract tumors? What is the standard treatment for high-risk UTUC? Are the oncological outcomes of open, laparoscopic and robotic approaches different?
	Should the bladder cuff be removed during radical nephroureterectomy? Why? Should template-based LND be performed in muscle-invasive upper urothelial carcinoma? If so, why? Is chemotherapy required after radical nephroureterectomy for upper urinary tract tumors? If so, which patients require it? Which chemotherapeutic agents are used after radical nephroureterectomy for upper urinary tract tumors? Do they increase survival? What does single post-operative intravesical instillation of chemotherapy do? In which patients should it be applied? What are the surgical options for patients with high-risk upper urinary tract urothelial tumors in the distal ureter? Can kidney-sparing treatment be performed in patients with high-risk upper urinary tract urothelial carcinoma? What are the risks this treatment? Do pembrolizumab and nivolumab have a role in the treatment of upper urinary tract urothelial carcinoma? If so, in which patients should they be used? Should we prefer laparoscopic surgery in patients with T3 and above? Can upper urinary tract urothelial carcinoma be treated with a percutaneous approach? If yes, which patients? What are the Platinum-eligible criteria for systemic treatment of upper urinary tract tumors? Is enfortumab vedotin used as a first-line treatment in metastatic upper urinary tract tumors? Which agents can be used in the first-line treatment of metastatic upper urinary urothelial carcinoma? Are there any benefit over each other? Is cisplatin-based combination chemotherapy effective in metastatic upper urinary urothelial carcinoma? If so, what benefits does it provide to the patient? Should cisplatin alone or cisplatin plus nivolumab be used in the treatment of metastatic upper urinary tract urothelial carcinoma? What treatments can be given to patients who cannot use cisplatin in the treatment of metastatic upper urinary tract urothelial carcinoma? Does radical nephroureterectomy provide survival benefits for patients with metastatic upper urinary tract urothelial carcinoma? What treatment options can be used to reduce symptoms in patients with metastatic upper urinary tract urothelial carcinoma? In patients without disease progression after 4 to 6 cycles of gemcitabine plus cisplatin or carboplatin, what treatment options can be offered to improve survival? In which patients with metastatic upper urinary tract urothelial carcinoma can erdafitinib be used? Does enfortumab vedotin increase overall survival in patients with metastatic upper urinary tract urothelial carcinoma? If so, in which group of patients?
Follow Up	At what intervals and with what tests should patients with low-risk upper urinary tract urothelial carcinoma be followed up after radical nephroureterectomy?
	At what intervals and with what tests should patients with high-risk upper urinary tract urothelial carcinoma be followed up after radical nephroureterectomy? At what intervals and with what tests should patients with low-risk upper urinary tract urothelial carcinoma be followed up after kidney-sparing management? At what intervals and with what tests should patients with high-risk upper urinary tract urothelial carcinoma be followed up after kidney-sparing management?
Frequently Asked Questions	What is upper urinary tract cancer and which organs does it affect? Who gets upper urinary tract cancer?
	Is upper urinary tract cancer contagious? What are the risk factors for upper urinary tract carcinoma? Is there a genetic predisposition to upper urinary tract cancer? What are the symptoms of upper urinary tract carcinoma? When should bleeding in urine (hematuria) be taken seriously? How are upper urinary tract tumors diagnosed? When are CT, MRI, cystoscopy, and ureterorenoscopy preferred for the diagnosis of upper urinary tract tumors? What is the role of urine cytology in the diagnosis of upper urinary tract tumors? What are the treatment options for upper urinary tract tumors? Who can undergo kidney-sparing (endoscopic) treatment for upper urinary tract tumors? What is radical nephroureterectomy? Is laparoscopic or robotic surgery possible for radical nephroureterectomy? Is lymph node dissection necessary in radical nephroureterectomy? In what cases is chemotherapy or immunotherapy applied for ureteral tumors? What do the stage and grade of the tumor mean?
